# Efficacy and safety of intense pulsed light direct eyelid application

**DOI:** 10.1038/s41598-022-17986-3

**Published:** 2022-09-16

**Authors:** María C. Martínez-Hergueta, Jorge L. Alió del Barrio, Mario Canto-Cerdan, María A. Amesty

**Affiliations:** 1 Division of Ophthalmology, Universidad Miguel Hernández, Alicante, Spain; 2 Ophthalmology Department, Hospital General de Elda, Alicante, Spain; 3 Cornea, Cataract and Refractive Surgery Unit, Vissum (Miranza Group), Alicante, Spain; 4 Oculoplastic Unit, Vissum (Miranza Group), Instituto Oftalmologico de Alicante, Avda de Denia s/n, Vissum, 03016 Alicante, Spain

**Keywords:** Outcomes research, Eyelid diseases

## Abstract

To describe the efficacy and safety of intense pulsed light (IPL) applied directly on the eyelids of patients with Meibomian gland dysfunction (MGD) without corneal shield protector. Observational retrospective single centre study where patients underwent 3 treatment sessions of IPL with 2 weeks of interval. The IPL was carried out with Lumenis OPT M22 with a double pass technique of 12 impacts on the infraorbital/lower eyelid region with the 15 × 35 mm guide light (step 1) and a double pass technique of 3 impacts over the upper eyelids with the 8 × 15 mm guide light (step 2). The follow up was conducted through Oculus Keratograph 5 M. 30 patients were enrolled in the study. Although there were no significant differences (*p* > 0.05), non-invasive tear break-up time, ocular redness, and OSDI questionnaire improved during the 3 IPL sessions. A significant improvement (*p* = 0.024) in the percentage of meibomian gland loss was also observed. Regarding tear meniscus, it was found similar measurements before and after treatment. No serious adverse effects were reported during the procedure or in subsequent follow-up. Preliminary results suggest that IPL therapy applied directly on the eyelids without corneal shield could be safe and effective in the treatment of MGD.

## Introduction

Intense Pulsed Light (IPL) has emerged as a new technique for Meibomian Gland Dysfunction (MGD) and evaporative dry eye management. IPL therapy consists in polychromatic pulses of non-coherent neither collimated light based on the principle of selective photothermolysis where the applied energy is absorbed by the chromophores of the human skin (haemoglobin, melanin and water) to generate heat that will destroy the tissue and ablate the blood vessels^1,2^. The equipment has an emission spectrum ranging from 500 to 1200 nm and mainly consists in a body, a handpiece and a light filter. The light, generated by a flash pulse xenon lamp, is concentrated by a reflector and its wavelength is filtered before its application on the skin. The use of infrared polychromatic light was first tested in 1976 for the treatment of vascular malformations^3^, it was described in detail in 1983^4^, and it was finally approved as a commercial medical device in 1994^5^. Since then, IPL therapy has been used for more than two decades in dermatology, but it was not until 2002 when it was observed that IPL treated patients with acne-rosacea improved their evaporative dry eye symptoms^6^.

Multiple non-randomized studies have been conducted suggesting a benefit in the dry eye population^7,8^, but there are not many clinical trials studying the use of IPL in evaporative dry eye and MGD, and there is still great discussion about its real efficacy and safety. A recently published systematic review^9^ found 3 randomized controlled clinical trials, with a total of 114 patients, evaluating the efficacy and safety of IPL for MGD^10–12^.

The first protocol proposed about the most appropriate and safe technique to perform IPL for evaporative dry eye treatment^13^, used energies range from 12 to 14 J/cm^2^ and applied the pulses of light on the periorbital region across the cheeks and nose with the aid of a facial eye shield or an opaque goggle to avoid light was absorbed by intraocular structures. Most authors have been using *these protectors*^11,14–16^ and most clinical guidelines recommend performing the technique under the use of a facial eye shield.

Despite this, it has already been proposed the possibility that direct application on the eyelid skin without facial shield and under adequate protection of the cornea and sclera could even improve further the results. Rong et al.^10^ applied 6 impacts of IPL pulses with fluences of 14–16 J/cm^2^ with the 8 × 15 mm guide light directly onto the eyelids using an ocular surface protector such as a Jaeger lid plate placed at the conjunctival sac.

But IPL is an increasingly demanded technique, and sometimes the protectors, especially those for the ocular surface, are uncomfortable for the patients and difficult to place. Toyos et al.^17,18^ evaluated the direct application of IPL without any ocular surface or facial protector and with a cylindrical guide light of 6 mm, observing that with 6 impacts of light and lowering to fluences of 10 J/cm^2^ for the upper eyelid, the technique was safe and also effective for the patients.

To make the technique simpler, *quicker and* more comfortable for the patient, the aim of our work is to demonstrate that performing the IPL with the conventional 8 × 15 mm guide light and 6 impacts on the upper eyelids without any facial or ocular surface protector is effective and safe for the treatment of MGD and reduce the possibility of adverse events related.

## Materials and methods

Observational retrospective single centre study conducted in our clinic, Vissum Miranza (Alicante, Spain). All patients provided written informed consent, and institutional review board approval from our institution was obtained with Board Approval Number IMO 201106_156. The study was performed in accordance with the tenets of the Declaration of Helsinki. We selected consecutive IPL treated patients between 2020 and 2021 meeting the inclusion criteria: (1) age of at least 18 years; (2) MGD or secondary evaporative dry eye^19^; (3) skin Fitzpatrick scale I-IV^20^. Exclusion criteria were: (1) acute intraocular inflammation; (2) skin Fitzpatrick scale V-VI (3) pregnancy; (4) piercings over the treated zone; (5) personal history of autoimmune diseases, epilepsy or prior herpes; (6) suspicious skin lesions; (7) photosensitivity; (8) treatment in the previous month with photosensitive drugs. All subjects were treated following the same IPL treatment protocol and underwent 3 IPL sessions performed by the same surgeon (MA) with 2 weeks of interval between each session (day 0, day 15 and day 30). Full ophthalmic examination was performed before and after each IPL session. All included patients completed the study.

### IPL procedure

**Figure 1:**
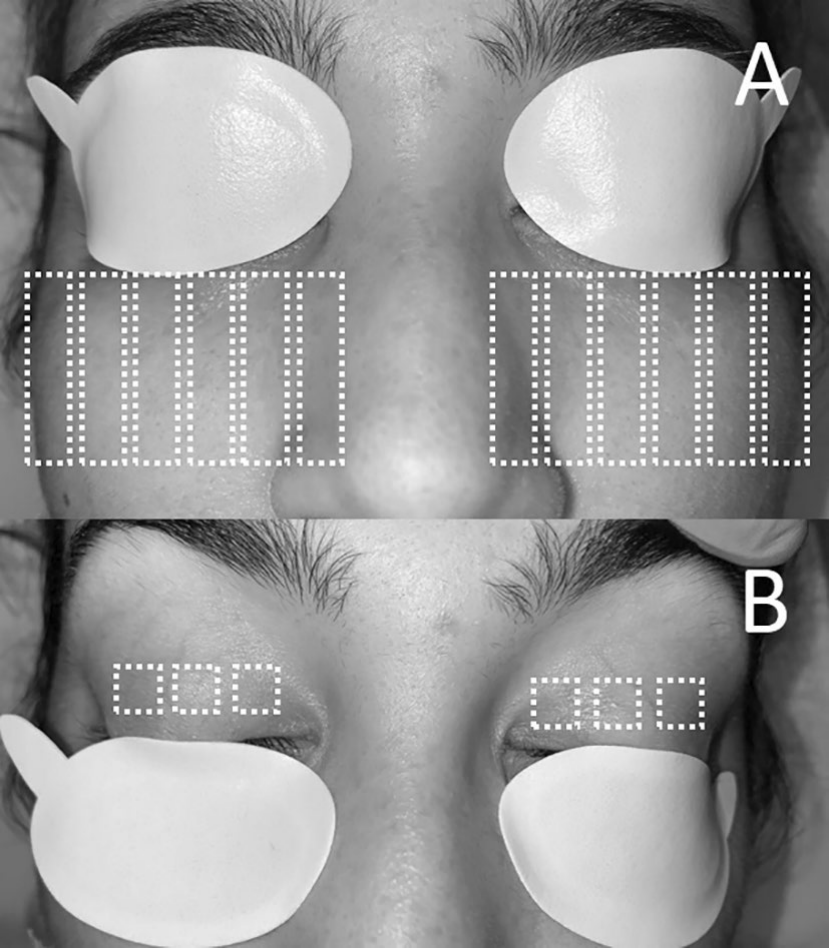
IPL protocol (**A**) Step 1. (**B**) Step 2.

Treatments were performed with the M22 Optima IPL (Lumenis, Israel) using a sapphire-cooled guide light of 15 × 35 mm and 8 × 15 mm, and the parameters were adjusted to the appropriate setting according to Fitzpatrick skin classification (Table 1) ^14^. Treatment was performed after facial cleaning, instillation of 0.1% tetracaine hydrochloride and 0.4% oxybuprocaine hydrochloride topical (Double anaesthetic, Alcon). The IPL protocol was performed in 2 steps: lower eyelids and upper eyelids. First (step 1), we treated the infraorbital and lower eyelid area covering upper lid and lashes with an adhesive facial eye shield (IPL-Eye Patch, Theia, USA) (Fig. 1A) and the 15 × 35 mm guide light. We applied a thick layer of cold ultrasound gel and we performed a double pass technique of 12 contiguous impacts from tragus to tragus across the cheeks and nose at 3 mm from the lower lid margin. Secondly (step 2), we removed the adhesive eye shield from the upper eyelid and placed it on the lower eyelid keeping the eyes closed, hiding the upper and lower eyelashes (Fig. 1B), and applying ultrasound gel on the upper eyelid. We asked patients to look down and we pulled from the brow upwards to tighten the upper eyelid skin. We performed two series of 3 overlaps impacts of IPL with the 8 × 15 mm guide light at 3 mm from the lid margin.

**Table 1:** Parameters used according to Fitzpatrick classification.

Fitzpatrick skin	Wavelength filter (nm)	Fluence (J/cm^2^)	Pulses	Duration (ms)	Delay (ms)
**Lower eyelids-Cheek-Nose**					
I	560	20	Triple	3	15
II	560	19	Triple	3	20
III	560	18	Triple	3	25
IV	590	17	Triple	3	30
**Upper eyelids**					
I	590	11	Triple	6	50
II	590	11	Triple	6	50
III	590	10	Triple	6	50
IV	590	10	Triple	6	50

Finally, we performed the expression of the meibomian glands using Collins meibomian gland forceps (German) immediately after the IPL treatment.

### Clinical assessment

Data collection before and 45 days after the enrolment (2 weeks after the last IPL therapy) included: (1) best corrected visual acuity (BCVA); (2) intraocular pressure (IOP); (3) slit-lamp examination (ocular surface, corneal or eyelid abnormalities, meibomian gland yield, Oxford scale of corneal fluorescein staining^21^, lens injury and fundus examination); (4) and Oculus Keratograph 5 M (K5M) (Oculus, Wetlzar, Germany) with Jenvis dry eye report (Jenvis Research Institute, Jena, Germany) including: (a) tear meniscus height (TM); (b) non-invasive tear break- up time (NI-BUT); (c) ocular redness according to the Jenvis grading scale; (d) infrared meibography; (e) Ocular Surface Disease Index (OSDI) questionnaire^22^. TM was qualitatively analysed based on its height, assuming a value of 1 as very high (≥ 0.35 mm); 2 as normal (0.20–0.35 mm); 3 as slightly reduced (0.15–0.20 mm) and 4 as low (≤ 0.15 mm). NI-BUT was also qualitatively analysed, considering 1 as above average (≥ 15 s); 2 as normal (11–15 s); 3 as short (7–11 s); and 4 as very short (< 7 s). *Ocular redness was classified based on the area percentage ratio between the vessels and the rest of the analyzed area*, *with the maximum ratio, according to the manufacturer of 4, so consider 1 as normal redness (0 to 1.5); 2 as mild redness (1.6 to 2.5); 3 as moderate (2.6 to 3.5) and 4 as severe (3.5 to 4).* Meibomian glands were analysed according to the degree of loss on the Jenvis grading scale: 1 for a loss under 33%; 2 for a loss between 33 and 66%, and 3 for a greater loss.

### Ethical approval and consent to participate

Ocular microsurgery institute approval obtained and written informed consent was obtained from all study participants.

### Consent for publication

The participants have consented the submission of data to the journal.

### Statistical analysis

All data was analysed using IBM SPSS Statistics for Windows version 22.0. Descriptive Statistics were expressed as means and standard deviations. After testing the normality of the variables with the Kolmogorov–Smirnov test, data before and after IPL was compared using the Wilcoxon signed-rank test. Correlations were evaluated with Spearman’s Rho correlation. Statistical differences were set at *p* < 0.05.

## Results

30 patients (60 eyes) were included in the study, of which 20 (66.6%) were women and 10 (33.3%) men. Mean age was 57.7 ± 15.9 years (range 28–85).

Most patients tolerated well the IPL sessions, and all patients were able to complete them. During the IPL procedure, 4 patients (13.34%) complained about pain and discomfort at some point, but by doubling the delay between pulses, these sessions could be finished without further complaints. No adverse events following IPL were reported in any patient, including intraocular inflammation, iris transillumination, lens injury, skin burns, ocular hypertension, fundus abnormalities or visual loss.

TM, NI-BUT redness and meibography results were qualitatively analysed as previously described (Tables 2, 3). No significant differences were found in the measurement of TM (*p* = 0.724) (Table 2). Statistically significant values were also not observed neither in NI-BUT nor in the ocular redness outcome (*p* > 0.05). However, while 37.29% of patients had a NI-BUT considered short or very short before IPL (2.07 ± 1.17), this was reduced to 33.33% at the end of follow-up (1.97 ± 1.15) (Tables 2, 3).

**Table 2:** Descriptive analysis of the Keratograph outcomes of the 60 eyes.

	Pre Treatment	Post Treatment		Pre Treatment		Post Treatment	
	Mean ± SD	Mean ± SD		Frecuency	Valid percent (%)	Frecuency	Valid percent (%)
Tear Meniscus (millimeters)	0.33 ± 0.14	0.38 ± 0.17	Very high	26	43.3	24	40
			Normal	29	48.3	30	50
			Slightly reduced	3	5	5	8.3
			Low	2	3,3	1	1.7
BUT (seconds)	13.60 ± 5.01	14.95 ± 5.71	Above average	28	47.46	31	51.7
			Normal	9	15.25	9	15
			Short	13	20.3	11	18.3
			Very short	10	16.9	9	15
Redness (vessels ratio)	1.48 ± 0.60	1.43 ± 0.55	Normal	36	60	37	61.7
			Mild	20	33.3	20	33.3
			Moderate	4	6.7	3	5
Meibography	1.59 ± 0.67	1.40 ± 0.74	No loss	1	1.7	2	3.3
			Less than 33%	27	45.8	39	65
			Less than 66%	26	44.1	12	20
			More than 66%	5	8.5	7	11.7

Tear Meniscus, BUT and ocular redness have been described as quantitative outcomes also.

**Table 3:** Statistical results of the Keratograph outcomes of the study and comparison of the Pre-Post IPL using the Wilcoxon paired samples test (*p* < 0.05).

	Pre treatment	Post treatment	*p*-value
	Mean ± SD	Mean ± SD	
Tear Meniscus	1.68 ± 0.73	1.72 ± 0.69	0.724
BUT	2.07 ± 1.17	1.97 ± 1.15	0.690
Redness	1.47 ± 0.62	1.43 ± 0.59	0.660
Meibography	1.59 ± 0.67	1.40 ± 0.74	0.024*
OSDI	32.03 ± 23.82	30.85 ± 21.41	0.880

We couldn’t find a statistically significant improvement in OSDI questionnaire (*p* = 0.888), despite OSDI score improved from 32.03 ± 23.8 pre-IPL to 30.85 ± 21.4 after 3 IPL sessions.

**Figure 2:**
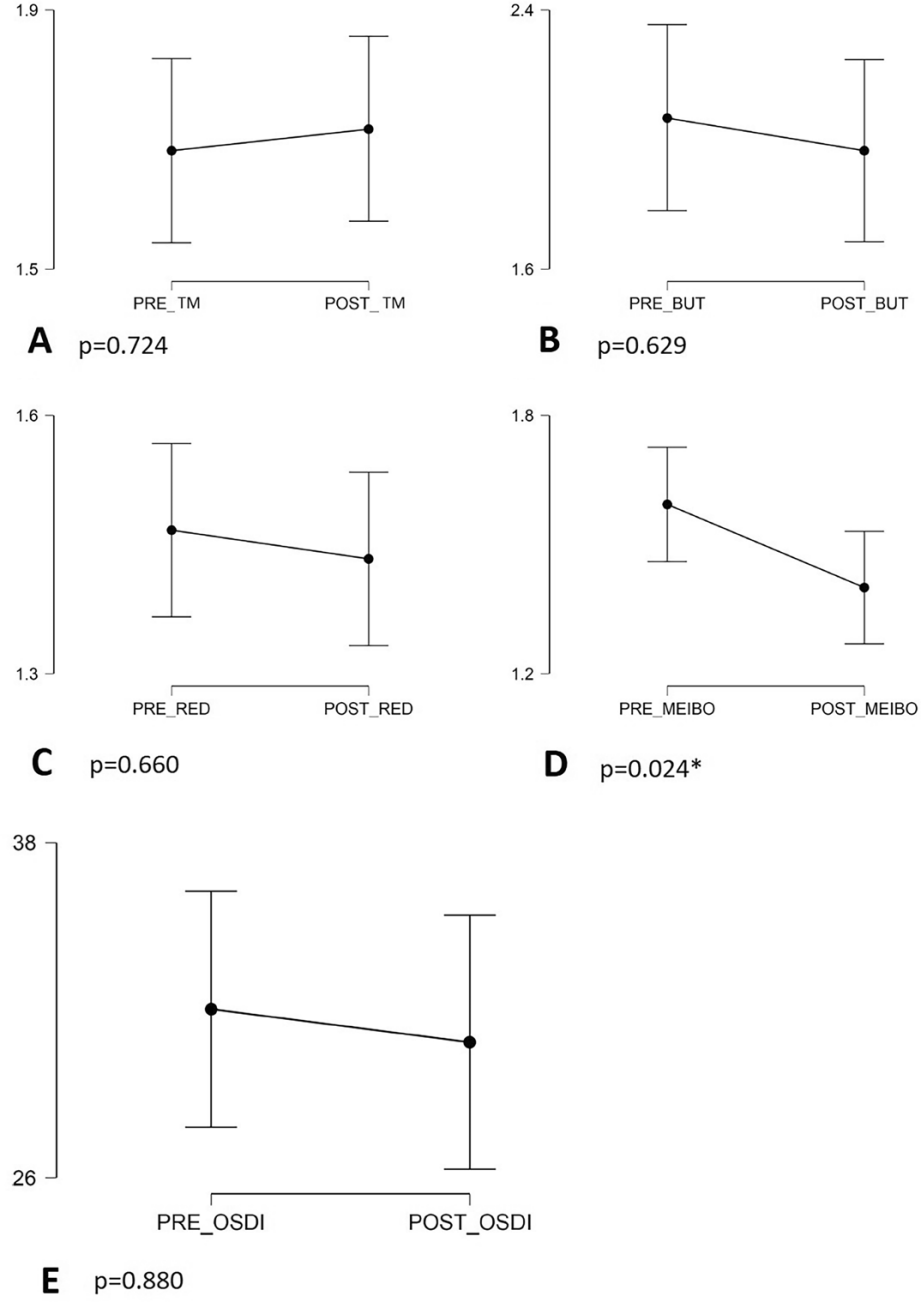
Time outcomes before and after treatment. (preTM, pre tear meniscus; postTM, post tear meniscus; preBUT, pre break-up time; postBUT, post break-up time; preRED, pre redness; postRED, post redness; preMEIBO, premeibography; postMEIBO, postmeibography).

**Figure 3:**
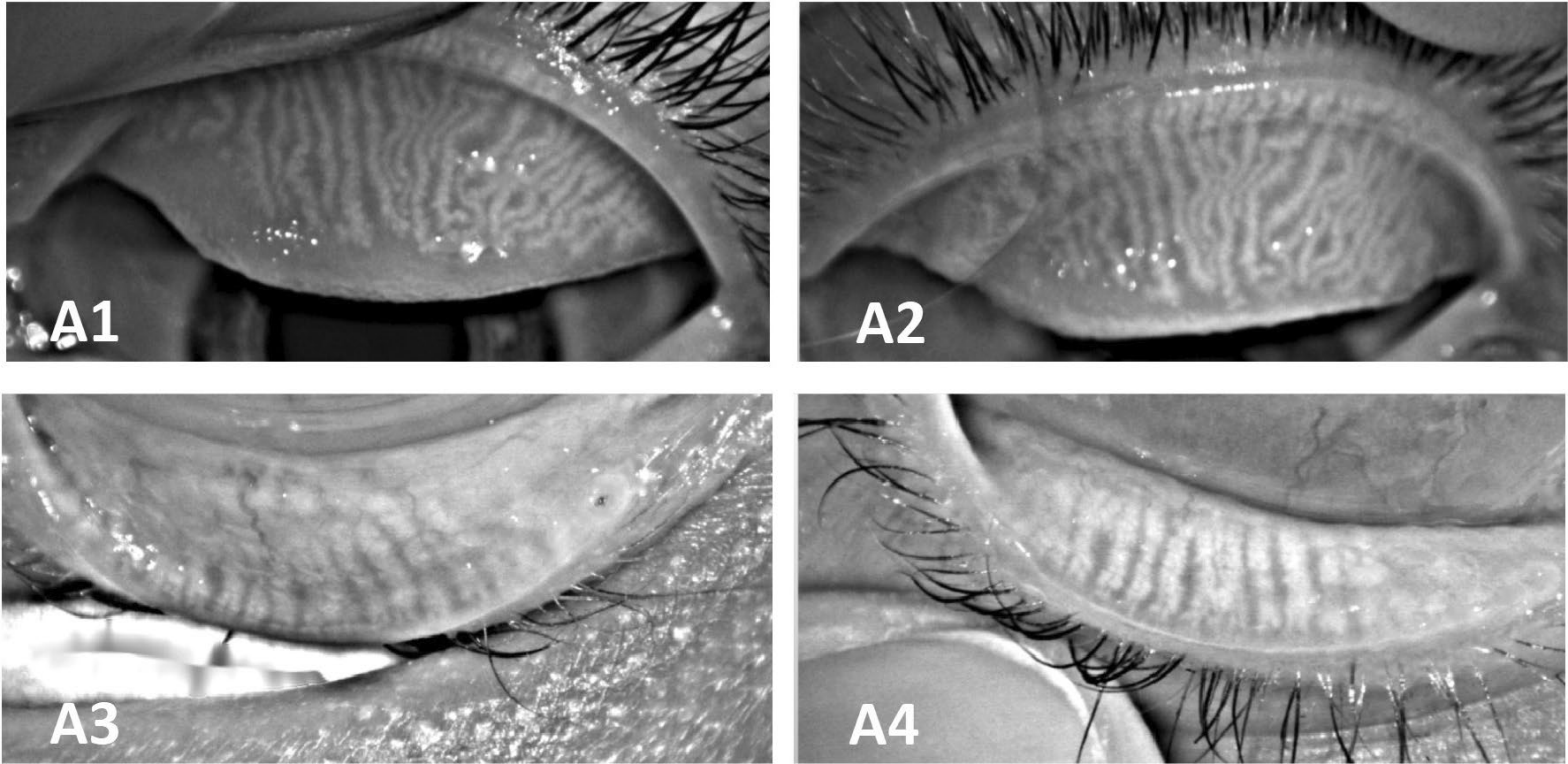
Meibography examination of the upper and lower eyelids before (A1, A3) and after (A2, A4) IPL showed that there is an improvement in the number of glands.

Meibography showed a statistically significant improvement after the 3 IPL sessions (*p* = 0.024). Pre-IPL data showed that 52.5% of study population had a percentage of Meibomian gland loss ≥ grade 2, while only 31.67% presented it at the end of the follow-up (Table 3 and Figs. 2, 3).

There was a direct correlation between age and redness before (*r* = 0.683, *p* < 0.001) and after treatment (*r* = 0.689, *p* < 0.001) (older patients presented greater ocular redness). We also found an inverse correlation between age and tear meniscus height before (*r* = − 0.354; *p* = 0.006) and after treatment (*r* = − 0.382; *p* = 0.003) (older patients had a smaller tear meniscus). No correlation with age was found for the rest of parameters (Table 4).

**Table 4:** Spearman rho correlation between age and Keratograph outcomes.

		Spearman’s rho	*p*-value
Tear Meniscus*	Pre treatment	− 0.354	0.006*
	Post treatment	− 0.382	0.003*
BUT	Pre treatment	0.084	0.529
	Post treatment	0.159	0.225
Redness*	Pre treatment	0.683	< 0.001*
	Post treatment	0.689	< 0.001*
Meibography	Pre treatment	− 0.056	0.675
	Post treatment	0.108	0.414
OSDI	Pre treatment	0.009	0.944
	Post treatment	− 0.234	0.089

Significant values are in italics.

## Discussion

IPL mechanism of action for dry eye disease is not entirely clear, although most authors propose that this therapy leads to events such as the destruction of superficial blood vessels with subsequent reduction of local inflammation, the liquefy of the meibum and antimicrobial, anti-inflammatory and antioxidant effects^23^.

During their evolution, IPL devices have been modified in order to reduce its side effects and expand its indications. The new generations allow filtering the wavelength spectrum by quartz or sapphire crystals towards greater lengths that impact on skin structures deeper in respect to the epidermis. The removable filters also allow to choose the wavelength needed for specific lesions. In the same way, a fractionation of the pulse energy has been carried out, and new cooling systems have been designed to avoid skin injury^1,2,23^. Among the side effects that may occur, the most frequent is pain during treatment application. This has been mitigated thanks to new refrigeration systems. Skin erythema and oedema have been reported hours or less commonly days after treatments, but both are transitory. Pigmentation changes and hypertrophic scars are uncommon^11,15,16,23^. Ocular complications have been described also when applying high fluences as 20 J/cm^2^. There are reports of anterior uveitis, iris transillumination defects and alterations that lead to synechiae and pupillary blockage with subsequent angle closure^24–26^.

Rong et al.^10^ used the 8 × 15 mm guide light and applied IPL pulses with the 560 nm filter and fluences of 14–16 J/cm^2^ directly onto the eyelids with a Jaeger lid plate and demonstrates that at this fluences and under corneal surface protection the treatment is effective and safe without ocular complications. Toyos et al ^17,18^ used the 6 mm cylindrical guide light with a 590 nm filter and fluences of 10 J/cm^2^ and they obtained promising results in terms of safety and efficacy. However, their assessments consisted in a clinician-measured tear breakup time and subjective questionnaires for dry eye symptoms 24 h prior the IPL treatment.

In our study, we have used the 8 × 15 mm guide light with fluences of 10–11 J/cm^2^ and the 590 nm filter onto the upper eyelids and we have evaluated its efficacy through a non-observer dependent test as the Oculus Keratograph 5 M. Safety was assessed through BCVA, IOP measurement, and slit-lamp examination to assess for abnormalities. No adverse effects were reported after our technique, as previously mentioned, so its direct application at low energies without a facial or ocular surface shield seems to be safe. Objective data regarding the efficacy showed an improvement in all the analysed parameters (except for the TM), but differences were statistically significant for the meibography, observing an improvement in the number and morphology of the meibomian glands. The 6 mm cylindrical guide light is likely to be able to concentrate energy more optimally in the area to be treated, even improving these outcomes.

Limitations of our study include its retrospective nature and the relatively small study sample. Further randomized control trials with larger sample are necessary to determine the optimal level of energy for each guide light type to assure safety, without the aid of a corneal shields, while keeping an adequate level of efficiency.

## Conclusions

To the authors’ knowledge, this is the first time that the follow-up of IPL procedure directly applied without corneal shield protector for MGD have been carried out by the Oculus Keratograph 5 M. In summary, although more studies are needed to determine if the 6 mm cylindrical handpiece can maintain previously reported levels of effectiveness, performing this procedure with the conventional guide and low-fluences has been proved to be safe, since there were no adverse effects, and shows objective meibography improvement and results of moderate efficacy in the NI-BUT, ocular redness and OSDI questionnaire.

## Data Availability

All data generated or analysed during this study are included in this published article [and its supplementary information files].

## Abbreviations

BCVA: Best corrected visual acuity
IOP: Intraocular pressure
IPL: Intense pulsed light
K5M: Oculus Keratograph 5 M
MGD: Meibomian gland dysfunction
NI-BUT: Non-invasive tear break- up time
OSDI: Ocular Surface Disease Index questionnaire
TM: Tear meniscus
